# *De Novo* transcriptome combined with physiological analyses revealed key genes for cadmium accumulation in Zhe-Maidong (*Ophiopogon japonicus*)

**DOI:** 10.3389/fpls.2022.1078330

**Published:** 2022-12-12

**Authors:** Qian Zhao, Qing Li, Shuting Lin, Ruijun Hou, Jianying Liu, Yachen Tao, Ye Li, Yifeng Zhou, Zihong Ye, Xiaoping Yu, Jun Huang

**Affiliations:** ^1^ School of Biological and Chemical Engineering, Zhejiang University of Science and Technology, Hangzhou, China; ^2^ College of Life Science, China Jiliang University, Hangzhou, China

**Keywords:** Zhe-Maidong (Ophiopogon japonicus), cadmium stress, cadmium accumulation and distribution, transcriptome, transporters

## Abstract

**Introduction:**

Cadmium (Cd) is a toxic heavy metal that severely threatens safe food production. Zhe-Maidong, a well-known Chinese traditional herbal medicine, is susceptible to Cd stress. However, the characteristics of Cd transformation and migration, as well as the regulatory system for genes conferring Cd accumulation of Zhe-Maidong, remains an essential issue to be addressed.

**Methods:**

Zhe-Maidong seedling growth in Cd-contaminated and uncontaminated soil was conducted for 90 days. The Cd concentration was determined by inductively coupled plasma-mass spectrometry, and the Cd^2+^ fluorescence probe detected Cd distributions. The root transcriptome of Zhe-Maidong was then evaluated using various Cd stress hydroponic treatments designated Cd-0, Cd-M, and Cd-H.

**Results and discussion:**

The enrichment factor (EF) value in the root was four times that of the leaves, indicating that the root has a high ability to absorb and accumulate Cd. The Cd^2+^ were mainly distributed in the root hair and the epidermis in both roots and leaves, revealing that the epidermal cells of roots may collect Cd^2+^ and also have an outstanding role in Cd^2+^ uptake. A total of 50 DEGs involved in Cd translocation and accumulation were identified. Among these DEGs, ANN, ABCC2/4, HMA1- 5, and CCX gene expression were positively correlated with EF-root, EF-leaf, EF-total, Cd-leaf, Cd-root, and Cd-plant, indicating their role in Cd transport and accumulation under Cd-stress. These data could be helpful in uncovering the Cd accumulation characteristics in Zhe-Maidong, as well as provide a bioinformatic foundation for investigations on finding gene functions and the screening of candidate genes related to Cd accumulation.

## 1 Introduction

Zhe-Maidong (*Ophiopogon japonicus* (L.f.) Ker-Gawl) is a prominent Chinese traditional herbal medicine that has been widely used in China for more than 2000 years (Liu et al., 2017). According to traditional Chinese medical theory, it replenishes the *yin*, enhances body fluid production, moistens the lung, and relieves heartburn (Yu et al., 2021). Zhe-Maidong contains a range of phytochemicals like as flavonoids, saponin, and polysaccharides, and it has antioxidation, anti-inflammation, immunomodulation, cardiovascular protection, and anticancer properties (Liu et al., 2017). In recent years, with the growth of the traditional Chinese medicine industry and the expansion of global commerce (Fu et al., 2021), the planting area of Zhe-Maidong has extended steadily. However, most farmers cultivate Zhe-Maidong in a dispersed manner, and soil and climate conditions were not considered before planting, resulting in poor quality and heavy metal residues in Zhe-Maidong (Jiang et al., 2018).

Rapid industrialization in China during the last three decades has resulted in widespread contamination of Cadmium (Cd) in agricultural soils (Wang et al., 2019a; Fu et al., 2021). Due to the high mobility, Cd is easily absorbed by plants, posing a severe threat to safe food production (Chihmin et al., 2021). Zhe-Maidong is primarily and widely planted in Zhejiang Province, and its grade quality is superior to all other *O. japonicus* produced in China. However, previous research has found that the rate of Cd concentrations exceeding the Chinese herbal medical limit is around 16.67% during 2003–2020 in Zhejiang province (Wang et al., 2019b; Chao et al., 2021). Moreover, Cd content detection on several batches of *O. japonicus* revealed that a portion of the product exceeded the threshold for the Cd limit (Wang et al., 2021). In light of the nutritional significance of Zhe-Maidong, it is urgent to comprehend the mechanism of cadmium absorption and transport to provide a crucial foundation for alleviating cadmium accumulation.

To date, numerous studies have been undertaken to investigate relationships between plant and soil Cd levels on the factors of the metal availability in soil, the root uptake processes, Cd distribution in plant organs, as well as mechanisms of Cd adsorption and essential genes for Cd transport in various plant species. Multiple pieces of research have demonstrated that Cd distribution in plants varies significantly between species and within a species, depending on the population or cultivar. (Florijn and Van Beusichem, 1993; Greger and Löfstedt, 2004; Laporte et al., 2015; Lewis et al., 2018; Huang et al., 2020; Zhang et al., 2022; Chi et al., 2022). In addition, several studies have explored the biological mechanisms of Cd accumulation in plants, emphasizing identifying the genes and proteins involved in Cd absorption, transport, storage, and detoxification in plant tissues. A number of genes were responsible for Cd uptake, transportation, and phloem loadings, such as IRT1 (iron-regulated transporter-ZIP family), natural resistance-associated macrophage protein (NRAMP), the heavy metal ATPase (HMA) transporters, and ABC transporters in rice, barley, *Arabidopsis thaliana*, and *Sedum plumbizincicola* (Sterckeman and Thomine, 2020). However, the characteristics of Cd transformation and migration, as well as the regulatory system for genes conferring Cd accumulation of Zhe-Maidong, remains an essential issue to be addressed.

We conducted different Cd treatments in the present study using two culture systems (soil cultivation and hydroponics). Initially, Zhe-Maidong growth in Cd-contaminated soil was employed to evaluate the regularities of transformation and migration, as well as the distribution of Cd in Zhe-Maidong tissues. Following that, RNA sequencing (RNA-Seq) techniques were used to identify candidate genes related to Cd accumulation in the root of Zhe-Maidong treated by hydroponic. And the expression of critical genes associated with Cd accumulation was further validated by quantitative real-time PCR (qRT-PCR). Finally, to screen the core genes closely related to Cd accumulation, we performed a correlation coefficient analysis on the relative expression of several essential genes and the Cd accumulation main feature of Zhe-Maidong under Cd-contaminated soil. These data could be helpful in uncovering the Cd accumulation characteristics in Zhe-Maidong, as well as provide a bioinformatic foundation for investigations on the finding of gene functions and the screening of candidate genes connected to Cd absorption and translocation.

## 2 Materials and methods

### 2.1 Plant materials and treatments

Zhe-Maidong was collected from Cixi, Zhejiang Province, PR China. All the samples were authenticated by Prof. Fangmin Cheng (Zhejiang University, Zhejiang, PR China). 1-year-old seedlings with uniformity development were selected. We only preserved 5-6 inner leaves, cut off the underground part at the stem’s base, and kept 10-12 cm of the leaves. Then the plants were conducted with different Cd^2+^ treatments.

Experiment I:

The contaminated soil (CS) and uncontaminated soil (UCS) were collected from Fuyang, Zhejiang province. As shown in **Table 1**, the Cd content of CS exceeds the national Grade II value for soil Cd levels in China (0.3 mg/kg). One hundred seedlings were randomly separated into two groups (CS treatment or UCS treatment) and then transplanted into 10 L plastic pots (two plants per pot) filled with the corresponding soil (CS or UCS). The pots were placed in a greenhouse with natural light conditions and an average daily temperature of 25°C to 28°C. And the plants were uniformly managed and usually irrigated every 2-3 days, depending on requirements. Plants were grown for 90 days before harvesting.

**Table 1:** Cd concentrations and physicochemical properties of tested soils.

Soil	Cd concentration (mg/kg)	Available Cd concentration (mg/kg)	Moisture content (%)	pH	Total nitrogen (g/kg)	Available phosphorus (mg/kg)	Available potassium (mg/kg)
CS	0.38 ± 0.03*	0.21 ± 0.01*	44.60 ± 0.05	5.65 ± 0.06	1.98 ± 0.19	26.75 ± 2.5	134.00 ± 10.83
USC	0.11 ± 0.01	0.11 ± 0.00	46.72 ± 0.1	5.88 ± 0.24	1.98 ± 0.29	25.50 ± 2.89	125.25 ± 5.50

CS and UCS indicated contaminated soil and uncontaminated soil, respectively. * Indicate significant differences at p< 0.05 level among different treatments.

Experiment II:

The obtained seedlings were initially pre-cultured on the artificial substrate for 15 days. For hydroponic experiments, seedlings with uniform morphology were selected. The seedlings were transplanted to 15 L plastic pots (12 plants per pot) containing 10L 1/2-strength Hoagland’s nutrient solution under daily conditions of 25°C to 28°C. Then the plants grown in different pots were randomly classified into three groups. The treatments were the control (Cd-0), 1 mg/L Cd (Cd-M) (the maximum permitted Cd content in soil for agriculture), and 10 mg/L Cd (Cd-H), with three replicate pots for each treatment. The pH of the nutrient solution was adjusted to pH 6.0 ± 0.1. Plants were cultured in a greenhouse under natural light conditions and with an average diurnal temperature of 25°C to 28°C. The nutrient solution was renewed every three days. After 30 days of treatments, the roots were sampled and immediately frozen in liquid nitrogen and then stored at −80°C for RNA extraction.

### 2.2 Cd concentration detection and calculation of enrichment factor and transfer factor

Dried samples were digested in concentrated nitric acid (HNO_3_) and hydrogen peroxide (H_2_O_2_) at 120°C by Digi-Block ED54 digestion system (LabTech, Wilmington, MA, USA). According to (Pan et al., 2021), the Cd concentration was determined by inductively coupled plasma-mass spectrometry (ICP-MS; XSeries 2, Thermo, Waltham, MA, USA). Three biological replicates were used in each experiment.

The enrichment factor (EF) indices were calculated as follows:


$${\text{EF}}_{\text{root}}={\text{C}}_{\text{root}}/{\text{C}}_{\text{soil}}$$

$${\text{EF}}_{\text{leaf}}={\text{C}}_{\text{leaf}}/{\text{C}}_{\text{soil}}$$

$${\text{EF}}_{\text{plant}}=\left({\text{C}}_{\text{root}}+{\text{C}}_{\text{leaf}}\right)/{\text{C}}_{\text{soil}}$$

The transfer factor (TF) indices were calculated as follows:


$$\text{TF}={\text{C}}_{\text{leaf}}/{\text{C}}_{\text{root}}$$

Where C_root_, C_leaf_, and C_soil_ (mg/kg) represent the metal concentration in the dried samples of root, leaf, and soil, respectively.

### 2.3 Intracellular Cd localization

Fresh samples were placed in a small box filled with an optimal cutting temperature compound (OCT), which was then frozen with liquid nitrogen. The tissue blocks were sliced by a frozen section machine (CM1950, Leica, USA) at 10 µm and placed on slides. Detection of Cd^2+^ distribution and its fluorescence intensity in developing anthers was performed according to previous studies (Xue et al., 2020). Frozen sections were rinsed with PBS three times before incubating in a Cd^2+^ probe (Life Leadmium™ Green AM dye) at 28°C in the dark for 10 min. The fluorescence signals were observed by using confocal microscopy (Eclipse NI, Nikon, Japan).

### 2.4 RNA extraction, library preparation, and sequencing

The total RNA was isolated from the roots of Zhe-Maidong using a Trizol Plant RNA Extraction Kit (Takara Bio, Japan). Utilizing the RNA Nano 6000 Assay Kit of the Bioanalyzer 2100, the total quantities and integrity of RNA were determined (Agilent Technologies, CA, USA). According to the manufacturer’s instructions, mRNA was enhanced with oligo(dT) beads and fragmented into small fragments. mRNA was reverse transcribed into cDNA using random primers (Illumina, Inc., San Diego, United States). Illumina NovaSeq 6000 equipment was used to sequence the cDNA library products in paired-end sequencing technology with read lengths of 150 bp by Novogene Co. (Guangzhou, China). Each experiment included three biological replicates.

### 2.5 Filtering of sequencing data and *de novo* assembly

Low-quality reads (Q-value ≤ 20) and reads containing more than 10% ambiguous ‘N’ bases were declared poor-quality reads and deleted, leaving the remaining clean reads. The clean reads were used for *de novo* assembly using Trinity software (v2.6.6). Contigs were clustered and constructed based on paired-end information. Trinity found extended non-terminal sequences of contigs, and the resulting sequences were designated as unigenes. All assembled unigenes were searched using BLASTX against public protein databases with an E-value of less than 1.0×10^-5^; the databases included the Nr (NCBI non-redundant protein database), Nt String (NCBI non-redundant nucleotide sequences), Pfam (Protein family), KOG/COG (Clusters of Orthologous Groups of proteins), Swiss-Prot (A manually annotated and reviewed protein sequence database), KO (KEGG Ortholog database), and GO (Gene Ontology).

### 2.6 Differential expression analysis

Differential expression analysis of two treatments (three biological replicates per condition) was performed using the DESeq2 R package (1.20.0). DESeq2 provides statistical routines for determining differential expression in digital gene expression data using a model based on the negative binomial distribution. The resulting P-values were adjusted using Benjamini and Hochberg’s approach for controlling the false discovery rate. The threshold for significantly different expressions was set as padj0.05 and | Log_2_ (foldchange)| > 1.

### 2.7 qRT-PCR analysis

The cDNA preparation was carried out as described by the instructions (TaKaRa, Japan). SYBR Green real-time PCR Master Mix reagent Kit was used for qRT-PCR (Toyobo, Osaka, Japan). Reactions were performed using the Bio-Rad CFX96 real-time system (Bio-Rad, USA) according to the manufacturer’s protocol. **Supplemental Table S1** lists the gene-specific primer pairs utilized in this study. Tublin (Cluster-21637.82800) expression was used to normalize the amplification of genes, and their relative expression levels were calculated using the 2 (-CT) method. The mean values and standard errors were calculated using three separate biological replicates in triplicate.

### 2.8 Statistical analysis

Data were presented as the means of three biological repetitions ± standard deviation. Data were analyzed by one-way analysis of variance (ANOVA) using SPSS 19.0 (IBM, Portsmouth, UK). Correlation effects were calculated by Origin software. Different letters and * indicate significant differences at p< 0.05 level among different treatments.

## 3 Results

### 3.1 Cd accumulation and distribution in Zhe-Maidong leaves and roots

**Figure 1:**
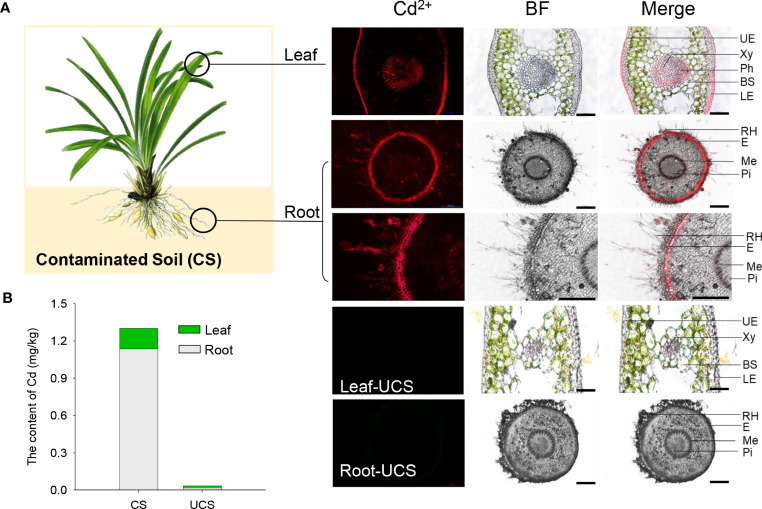
Analyses of Cd accumulation and distribution in Zhe-Maidong. **(A)** The distribution of Cd^2+^ in Zhe-Maidong leaves and roots using Leadmium Green AM dye. BF, bright field; Merge, the overlap of Cd^2+^ fluorescence and bright field. For each treatment, images from three technical replicates for three biological repeats were collected. UE, upper epidermis; Xy, xylem; Ph, phloem; BS, bundle sheath; LE, lower epidermis; RH, root hair; E, epiblema; Me, metaxylem; Pi, pith. Bars = 100 µm. **(B)** The Cd content in different tissues of Zhe-Maidong. CS, contaminated soil; UCS, uncontaminated soil.

According to the results presented in **Figure 1** and **Table 2**, the Cd content in the root and leaf tissues of Zhe-Maidong was significantly increased after being treated with cadmium-contaminated soil (CS). And the rise in Cd content was markedly more significant in the root than in the leaf. The Cd content of Zhe-Maidong roots and leaves was 1.14 mg/kg and 0.16 mg/kg, respectively, when the soil Cd concentration was 0.375 mg/kg and the available Cd concentration was 0.207 mg/kg after 90 days of CS treatment. The enrichment factor (EF) was increased significantly with the CS treatment, especially in the root. The total EF of Zhe-Maidong under CS treatment was 2.34, of which the EF of roots was as high as 1.88, while the EF of leaves was 0.46 (**Table 2**). The EF value of the roots was almost four times higher than that of the leaves. It was noteworthy that the transfer factor (TF) was significantly lower in the CS treatment than in the UCS, which was mainly attributed to the extremely low and similar Cd content deposited in the roots and leaves of Zhe-Maidong in the UCS.

**Table 2:** Cd contents in Zhe-Maidong with different soil.

Soil	Cd content (mg/kg)			EF			TF
	Root	Leaf	Plant	Root	Leaf	Total	
CS	0.68 ± 0.01*	0.17 ± 0.01*	0.85 ± 0.02*	1.88 ± 0.05*	0.46 ± 0.04*	2.34 ± 0.09*	0.24 ± 0.02
UCS	0.03 ± 0.00	0.02 ± 0.00	0.04 ± 0.00	0.25 ± 0.03	0.15 ± 0.01	0.40 ± 0.04	0.62 ± 0.07*

CS and UCS indicated contaminated soil and uncontaminated soil, respectively. * Indicate significant differences at p< 0.05 level among different treatments.

Cd distributions in the leaf and root tissues were detected by the Cd^2+^ fluorescence probe, as shown in **Figure 1**. The result showed that the preferential Cd^2+^ fluorescence in the leaves was distributed to the upper epidermis, xylem, phloem, and lower epidermis, while the Cd^2+^ in the roots were mainly distributed in root hair, epiblema, and metaxylem. In addition, we found that the intensity of Cd^2+^ fluorescence was most noticeable in the hair and epidermis of the root, followed by the epidermis, xylem, and phloem in the leaf. It was suggested that these tissues/cells were significant sites for the Cd^2+^ uptake and accumulation in Zhe-Maidong.

### 3.2 Sequencing and *de novo* transcriptome assembly

**Figure 2:**
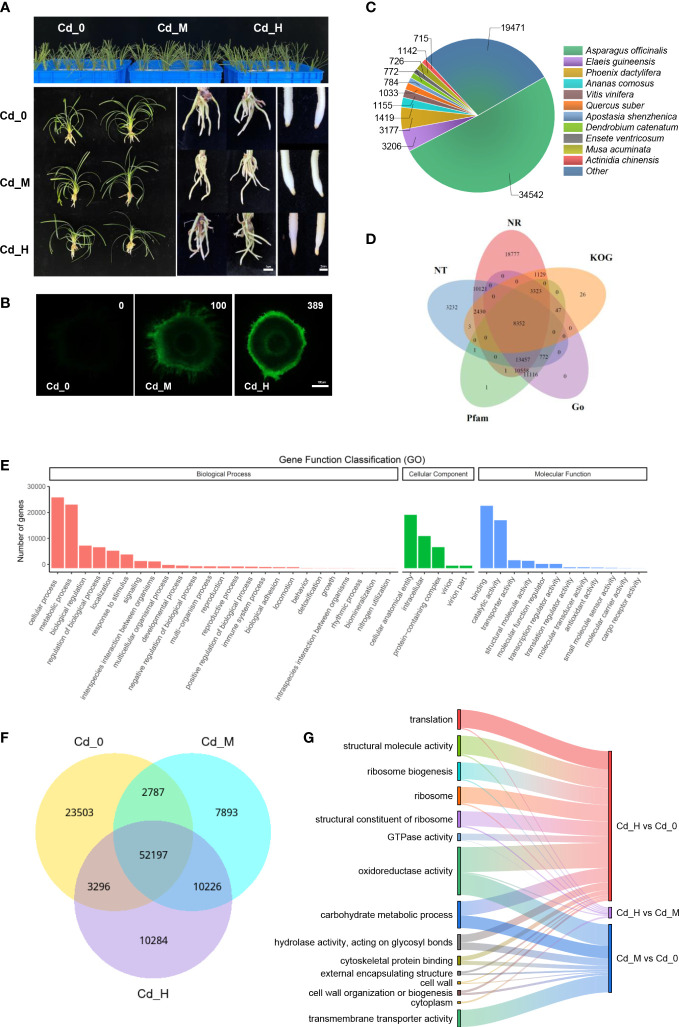
The phenotype and DEGs analysis of Zhe-Maidong exposed to three different Cd concertation. **(A)** The phenotype of Zhe-Maidong exposed to three different Cd concertation by hydroponics. The treatments were control (Cd-0), 1 mg/L Cd (Cd-M), and 10 mg/L Cd (Cd-H). **(B)** The distribution of Cd^2+^ in Zhe-Maidong leaves and roots using Leadmium Green AM dye. For each treatment, images from three technical replicates for three biological repeats were collected. The relative fluorescence intensities of Cd-0, Cd-M, and Cd-H were 0, 100, and 389, respectively. Bars = 100 µm. **(C)** Species distribution of homologous proteins of unigenes. The different color blocks represent different species. **(D)** Distribution of functionally annotated unigenes in the Go, KOG, NR, NT, and Pfam databases. **(E)** Functional annotation of unigenes based on Gene Ontology (GO) categorization. **(F)** DEGs under different Cd stress in Zhe-Maidong. **(G)** Go enrichment analysis of DEGs following the Cd stress treatment. The most significant 15 terms were chosen to be displayed.

Various Cd stress hydroponic treatments, designated Cd-0, Cd-M, and Cd-H, were performed to evaluate the root transcriptome of Zhe-Maidong under Cd stress (**Figure 2A**). There were no discernible differences in the biomass, morphology, plant length, and so on among Cd-0, Cd-M, and Cd-H (data not shown). However, under Cd stress (Cd-M and Cd-H), the root tips of Zhe-Maidong turned somewhat yellow, indicating an unhealthy situation. The Cd^2+^ fluorescence was detectable in the Zhe-Maidong roots subjected to various Cd stress hydroponic treatments with the intensity order Cd-H > Cd-M (**Figure 2B**).

Total RNA was isolated from the roots and subjected to various Cd stress hydroponic treatments to determine RNA sequencing and then performed on the Illumina NovaSeq 6000 equipment. After filtering sequencing data, a total of 212,557,374 clean reads of 9 samples were retained, with more than 92% Q30 bases acquired (**Supplemental Table S2**). The assembled unigenes were searched against several databases, including NR, NT, KOG, Pfam, and Go by BLAST. There were 68,148 (40.43%), 38,358 (22.76%), 47,628 (28.25%), 15,310 (9.08%), and 47,625 (28.25%) unigenes annotated by the NR, NT, Pfam, KOG, and Go databases, respectively (**Figure 2C**). Compared with the other databases, the NR database presented the highest proportion of annotations. Overall, the unigene sequences exhibited the most similar BLASTx matches to gene sequences from *Asparagus officinalis* (34,542), followed by *Elaeis guineensis* (3,206), *Phoenix dactylifera* (3,177), *Ananas comosus* (1,419), *Vitis vinifera* (1,155), *Musa acuminata* (1,134), and so on (**Figure 2D**).

### 3.3 Annotation and classification of Zhe-Maidong unigenes

GO analysis was used to classify the functions of the Zhe-Maidong unigenes. There were 183,939 unigenes successfully classified into 42 functional groups, which were further subdivided into three core GO categories: biological processes, cell components, and molecular function (**Figure 2E**). The dominant subcategories of the classified genes included ‘cellular process’ (26,640) and ‘metabolic process’ (23,932) in the ‘Biological processes’ category; ‘cellular anatomical entity’ (20,070), ‘intracellular’ (8,171) and ‘protein-containing complex’ (7,901) in the ‘Cellular Component category; ‘binding’ (23,483) and ‘catalytic activity’ (18,043) in the ‘Molecular Function’ category. A detailed Venn diagram depicts the number of transcripts shared by different Cd treatments (Cd-0, Cd-M, and Cd-H) of Zhe-Maidong (**Figure 2F**). The Venn diagram illustrates the effect of Cd stress on Zhe-Maidong is very complex, with 52,197 transcripts commonly expressed among different Cd treatments. There were also numerous specific transcripts expressed at Cd-0, Cd-M, or Cd-H.

All test samples were divided into three comparisons (Cd-H vs. Cd-0, Cd-H vs. Cd-M, and Cd-M vs. Cd-0), and each with upregulated and downregulated genes. In total, 24,222 differentially expressed genes (DEGs) (12,146 upregulated and 12,076 downregulated), 21,987 DEGs (10,679 upregulated and 11,308 downregulated), and 481 DEGs (319 upregulated and 162 downregulated) were found between Cd-H vs. Cd-0, Cd-H vs. Cd-M, and Cd-M vs. Cd-0, respectively (**Table 3**). Then, all differentially expressed genes (DEGs) following the Cd stress treatment were annotated by Go enrichment analysis. The most significant 15 terms were chosen to be displayed (**Figure 2G**). Among all DEGs from Cd-H vs. Cd-0, Cd-H vs. Cd-M, and Cd-M vs. Cd-0 were substantially enriched in oxidoreductase activity, carbohydrate metabolic process, structural molecule activity, and transmembrane transporter activity.

**Table 3:** Total number of differentially expressed genes (DEGs).

Compare	All	Up	Down	Threshold
Cd-H vs. Cd-0	24,222	12,146	12,076	DESeq2 padj<0.05 |Log_2_FoldChange|>1
Cd-M vs. Cd-0	21,987	10,679	11,308	DESeq2 padj<0.05 |Log_2_FoldChange|>1
Cd-H vs. Cd-M	481	319	162	DESeq2 padj<0.05 |Log_2_FoldChange|>1

### 3.4 DEGs involved in Cd translocation and accumulation

**Figure 3:**
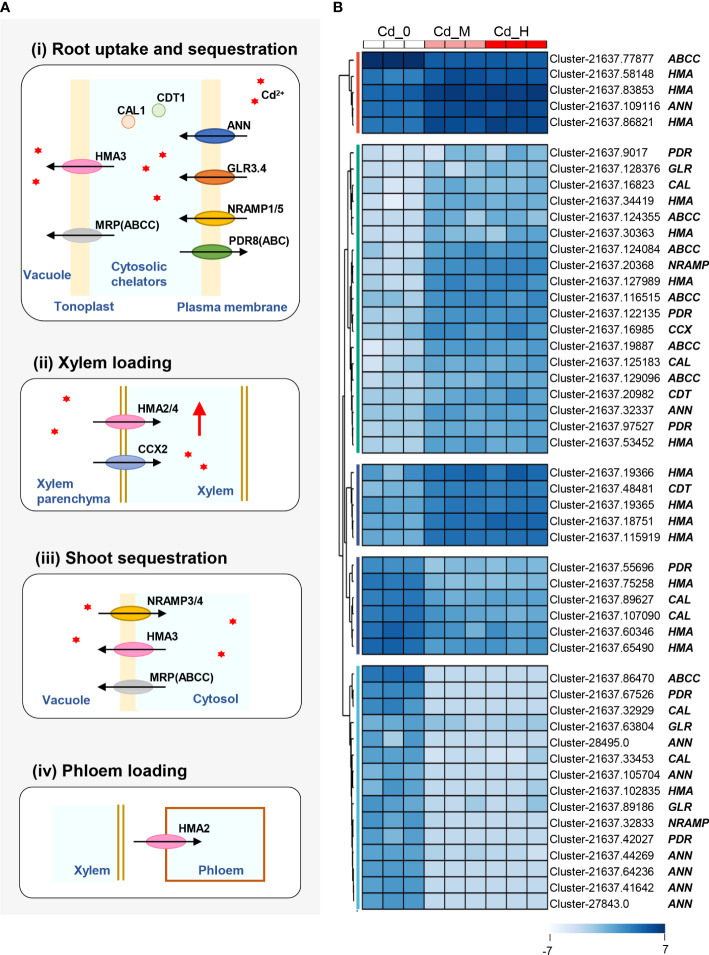
The DEGs of transporters/proteins involved in Cd accumulation. **(A)** The brief diagram of the transporters/proteins involved in Cd accumulation. **(B)** DEGs of transporters/proteins involved in Cd accumulation. The expression levels of DEGs were recorded as Log_2_ (FPKM). Cd^2+^was symbolized by red *.

To better understand the DEGs related to Cd accumulation of Zhe-Maidong and their corresponding functions, we illustrated a brief diagram of the transporters/proteins involved in Cd accumulation according to previous studies (Takahashi et al., 2012; Song et al., 2017; Sterckeman and Thomine, 2020) (**Figure 3A**), including root uptake and sequestration, xylem loading, shoot sequestration, and phloem loading. These processes are mediated by several proteins/transporters, such as natural resistance-associated macrophage protein (NRAMP), glutamate receptors (GLR), annexin (ANN), ATP binding cassettes (PDR/ABC), Cys-rich peptide (CDT), defensin-like protein (CAL), multidrug resistance-associated protein (MRP/ABCC), P18-type metal transporter ATPase (HMAs), and cation/calcium (Ca) exchanger (CCX).

Based on the Cd accumulation pathway in higher plants (**Figure 3A**), 50 DEGs involved in Cd uptake, transport, and accumulation were identified at a threshold of |Log_2_(foldchange)| > 2 with padj<0.05 in the comparisons of Cd-H vs. Cd-0 and Cd-M vs. Cd-0 (**Figure 3B**). These DEGs were found to be sequence similarity to the 9 family genes associated with Cd accumulation: *NRAMP, GLR, ANN, PDR, CDT, CAL, MRP, HMA*, and *CCX*, which can be divided into 5 groups based on their expression patterns. Four genes from the first group (excluding MRP), all 19 genes in the second group, and five genes in the third group were considerably upregulated by Cd stress treatments (Cd-M and Cd-H). Among all the upregulated genes, there were eleven *HMA* genes, five *MRP* genes, three *PDR* genes, two *CAL* genes, two *CDT* genes, two *ANN* genes, one *GLR* gene, and one *CCX* gene. In contrast, 20 DEGs were significantly downregulated in the fourth and fifth groups after Cd exposure. Furthermore, we found that DEGs expression patterns under moderate Cd exposure (Cd-M) were highly comparable to those under high Cd stress (Cd-H) (**Figure 3B**).

To validate the RNA-seq results, qRT-PCR was utilized to quantify the expression of 7 DEGs associated with Cd accumulation. The qRT-PCR results of these DEGs were mostly compatible with the RNA-seq data, as shown in **Supplemental Figure S1**. The correlation coefficient (R^2^ = 0.8628) indicated that the DEGs expression pattern obtained by RNA-seq and qRT-qPCR results were in good concordance, which supports the reliability of the RNA-seq data.

### 3.5 Correlation coefficient analysis between Cd accumulation and the expression of Cd accumulation-related genes

**Figure 4:**
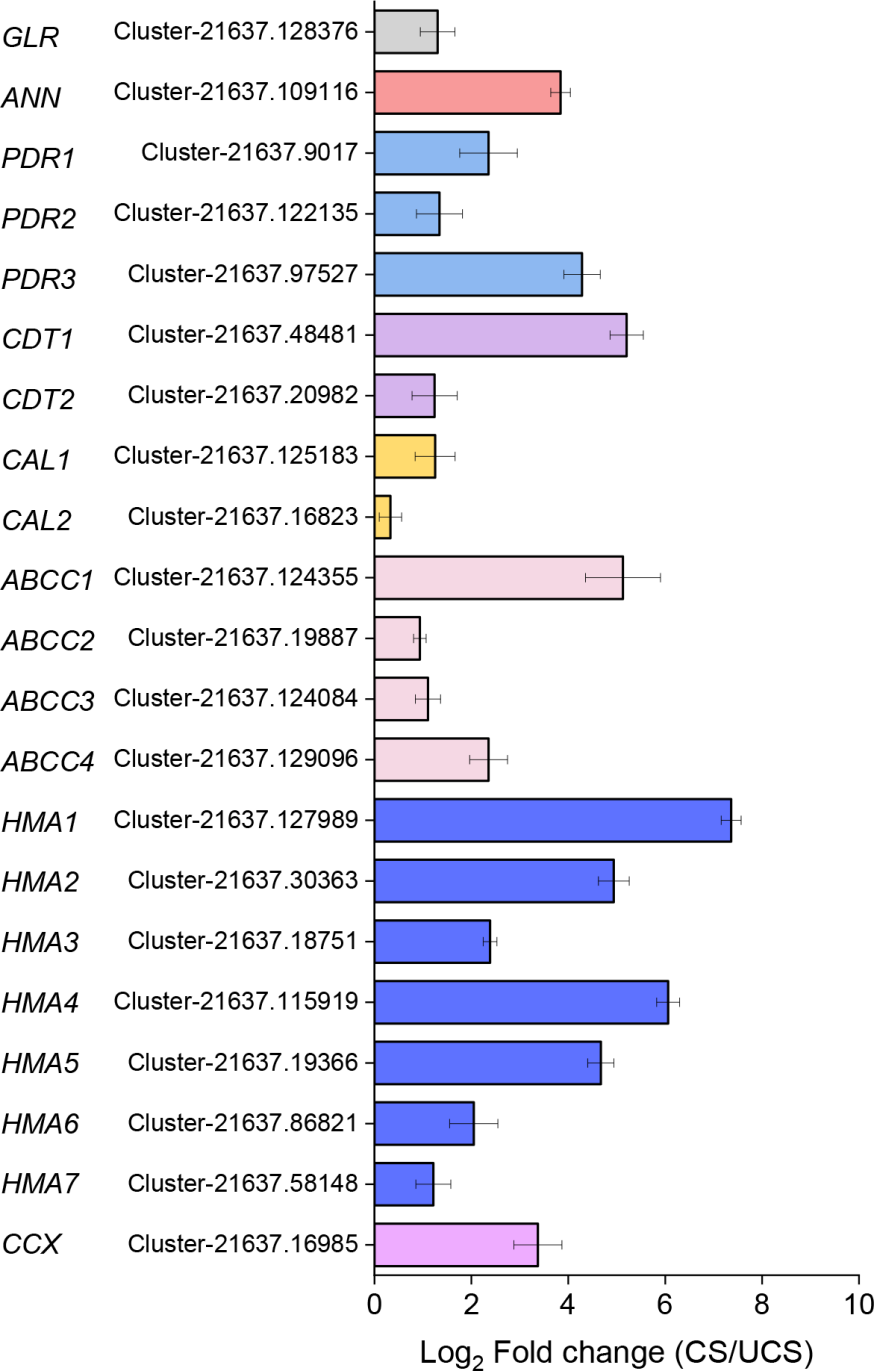
The relative gene expression levels of transporters/proteins involved in Cd accumulation under CS treatment as determined by qRT-PCR.

**Figure 5:**
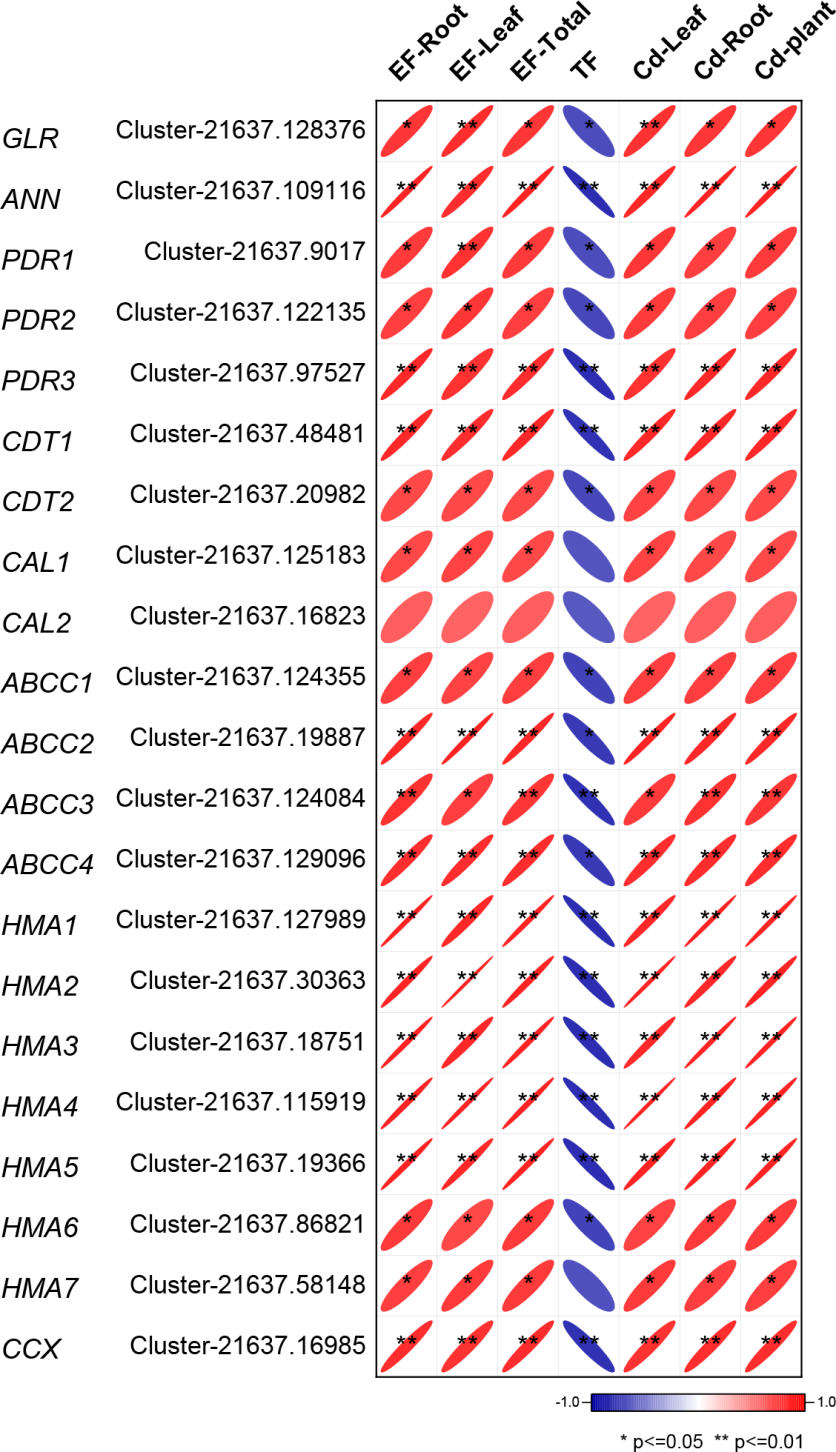
The correlation coefficient between Cd accumulation and the relative gene expression. * and ** denote statistically significant differences at the p<0.05 and p<0.01 levels, respectively.

We measured the relative expression levels of the obtained 21 genes that have the prediction cDNA sequence by RNA-seq in CS and UCS to determine their roles in Cd accumulation (**Supplemental Table S1**). As shown in **Figure 4**, all 21 genes were significantly upregulated when CS was compared to UCS. Under CS treatment, the relative gene expression levels of *ANN*, *PDR3*, *CDT1*, *ABCC1*, and *HMA1/2/4/5* increased the most. Then, we performed a correlation coefficient analysis between Cd accumulation (EF-root, EF-leaf, EF-total, TF, Cd-leaf, Cd-root, and Cd-plant) and the related gene expression (**Figure 5**). The results showed that the gene expressions of *ANN, PDR3, CDT1, ABCC2/4, HMA1-5*, and *CCX* were statistically and positively correlated with EF-root, EF-leaf, EF-total, Cd-leaf, Cd-root, and Cd-plant in the root of Zhe-Maidong under Cd-stress. Therefore, we inferred that higher gene expressions of *ANN, ABCC2/4, HMA1-5*, and *CCX* in the root of Zhe-Maidong under the Cd-stress were strongly responsible for Cd transport and accumulation. Interestingly, most gene expression levels were negatively correlated with TF coefficients, which we hypothesize is due to the fact that Cd accumulation in *Ophiopogon japonicus* roots was significantly higher than in leaves, causing TF values in plants with high Cd accumulation to be reversed.

## 4 Discussion

Previous studies reported that heavy metal contents in herbs whose roots are used as medicinal parts are higher than those in whole-grass herbs (Wang et al., 2019b). The root is the main organ for heavy metal uptake (Pál et al., 2006). In our present study, the Cd content of Zhe-Maidong roots was much more than that in leaves after exposure to CS for 90 days (**Figure 1**, **Table 2**). When exposed to CS, the EF value in the root was four times that of the leaves, whereas the TF value was only approximately 0.24. As a result, we suggested that the root can absorb and accumulate Cd while possessing a poor capacity for Cd transport from roots to leaves (**Table 2**). This finding was similar to recent studies on rice and 60 kinds of Chinese herbal medicines (Wang et al., 2019b; Li et al., 2022), which found that Cd is rarely translocated to aerial tissues and primarily accumulates in the roots. It could be attributable to Cd retention during long-distance transport from roots to leaves of Zhe-Maidong. Previous studies suggested that Cd is accumulated in the roots among most plants, which serves as a protective mechanism against Cd toxicity in whole plants (Zhou et al., 2017). However, for Zhe-Maidong, whose main edible parts are root organs, the massive accumulation of cadmium in the roots poses a threat to human health.

Cd subcellular distribution has been shown to be intimately related to tolerance mechanisms (Lu et al., 2017). Our result showed that the intensity of Cd^2+^ fluorescence was most noticeable in the root hair, as well as the epidermis in both roots and leaves (**Figure 1**). The root hairs are the most active regions for absorbing ions from the soil. These structures may facilitate the absorption of the majority of Cd from the soil (Sterckeman and Thomine, 2020). Similar characteristics have been detected in the root of various plants, such as rice (Chen et al., 2018) and sunflowers (Laporte et al., 2013). These findings can be related to the increased activity of transport systems near the root hair, where they may be expressed at higher levels and/or benefit from additional energy (Chen et al., 2018). Subsequently, Cd^2+^ entry into the root epidermis layer through the apoplast pathway (adsorption process) or the symplast pathway (through the appropriate ion channels) (Song et al., 2017; Sterckeman and Thomine, 2020). Cd accumulation in the epidermal cell layer was also identified in *Zea mays* seedlings grown in heavy metal-polluted soils, which is consistent with our test results (Seregin and Kozhevnikova, 2004). Previous studies revealed that the epidermal cell layer in plants is thought to be an absorbing tissue because it contains a more active system of membrane transporters (Sridhar et al., 2005). Thus, we proposed that the epidermal cells of roots in Zhe-Maidong not only have the potential to accumulate Cd^2+^, but also have an excellent feature for ion uptake. This also explains why the intensity of cadmium fluorescence in root epidermal cells is considerably higher than in leaf epidermal cells.

Transporters are necessary for the Cd translocation and accumulation processes (Sterckeman and Thomine, 2020) (**Figure 3**). In this study, RNA-Seq techniques were used to identify candidate genes related to Cd translocation and accumulation in the root of Zhe-Maidong treated by hydroponic. In the comparisons of Cd-H vs. Cd-0 and Cd-M vs. Cd-0, a total of 50 DEGs involved in Cd translocation and accumulation were identified at a threshold of |Log_2_ (foldchange)| > 2 with padj<0.05 (**Figure 3B**). Among all the DEGs, upregulated genes included eleven *HMA* genes, five *MRP* genes, three *PDR* genes, two *CAL* genes, two *CDT* genes, two *ANN* genes, one *GLR* gene, and one *CCX* gene. There were 20 DEGs significantly downregulated in the fourth and fifth groups after Cd exposure (**Figure 3B**). Additionally, a total of 21 predicted cDNA sequences by RNA-seq were used to determine their roles in Cd accumulation in the root of Zhe-Maiddong under CS for 90 days. According to **Figure 4**, the gene expressions of *ANN, ABCC2/4, HMA1-5*, and *CCX* were statistically and positively correlated with EF-root, EF-leaf, EF-total, Cd-leaf, Cd-root, and Cd-plant in Zhe-Maidong under Cd stress. These findings suggest that *ANN, ABCC2/4, HMA1-5*, and *CCX* genes were strongly responsible for Cd transport and accumulation in the root of Zhe-Maidong under Cd stress. In contrast to CAX, HMA, and ABCCs, the PDR8 protein (pleiotropic drug resistance) belongs to a wide family of ATP-binding cassette (ABC) transporters that are prominent in the root hairs and epidermis and have been linked to Cd efflux (Kim et al., 2007). Furthermore, *OsCDT1* may restrict cellular Cd accumulation by limiting Cd uptake by chelating the metal at the cellular surface, therefore improving Cd tolerance (Kuramata et al., 2009). In this paper, PDR3 (Cluster-21637.97527) and CDT1 (Cluster-21637.48481) gene expression levels were drastically upregulated in response to Cd stress, showing that they play an essential role in avoiding Cd stress and promoting Cd tolerance.

**Figure 6:**
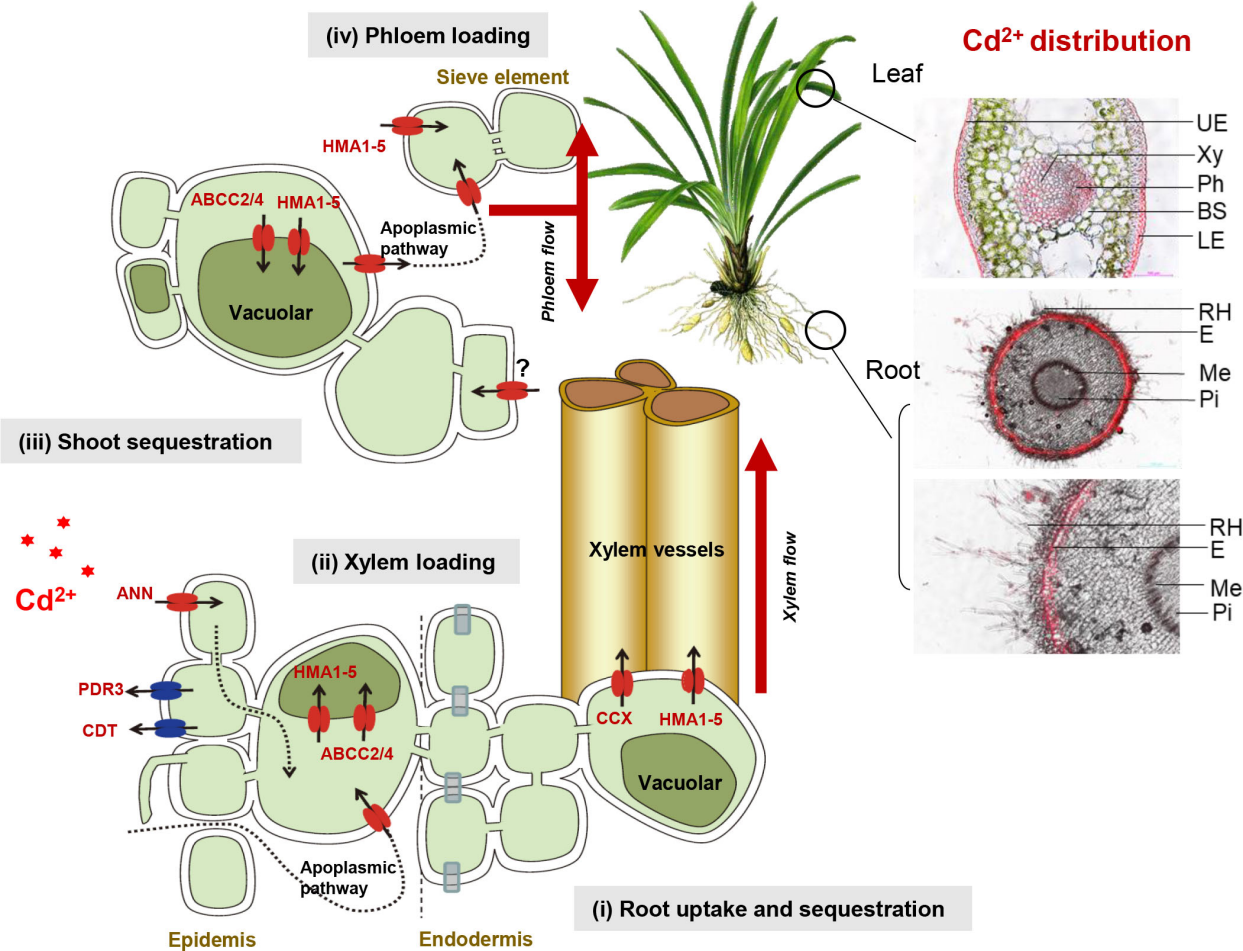
A schematic model of Cd uptake and accumulation in Zhe-Maidong. Cd^2+^ from the soil can diffuse or be carried passively by water flow in the apoplast of the root cortex (apoplastic pathway). ANN plays a vital role in Cd uptake in Zhe-Maidong. HMA1-5 could bind Cd and induce Cd^2+^ influx from the cytoplasm to the vacuole. In addition, HMA transporters also play an essential role in loading Cd in xylem vessels and phloem loading. MRP transporters (ABCC) were responsible for the influx of PC-Cd complexes into the vacuole. The PDR8 and CDT1 may be crucial in Cd efflux, in contrast to CAX, HMA, and ABCCs.

Based on our findings, we developed a schematic model of Cd accumulation in Zhe-Maidong (**Figure 6**). Cd^2+^ from the soil can diffuse or be carried passively by water flow in the apoplast of the root cortex (apoplastic pathway). Furthermore, Cd^2+^ entrance might occur through Ca^2+^ channels in root cell plasma membranes, which are permeable to a broad range of monovalent and divalent cations (Chen et al., 2018). Here, we supposed that ANN (Cluster-21637.109116) plays a vital role in Cd uptake in Zhe-Maidong. The study on rice indicates that the Ca^2+^ channels *OsANN4* can mediate Cd uptake in rice roots after exposure to 20 μM Cd (Chen et al., 2018). In plants, the vacuole is the primary organelle for Cd storage. HMA1-5 (Cluster-21637.127989, Cluster-21637.30363, Cluster-21637.18751, Cluster-21637.115919, Cluster-21637.19366) and ABCC2/4 (Cluster-21637.19887, Cluster-21637.129096) were considered to bind Cd and induce its influx from the cytoplasm to the vacuole in roots. HMA transporters also play an essential role in the loading of Cd in xylem vessels and phloem loading. Members of the HMA family have been identified in a variety of plant species, including *A. thaliana* (Wong and Cobbett, 2009), rice (Treesubsuntorn and Thiravetyan, 2019), barley (Zhang et al., 2021), and wheat (Kintlová et al., 2021). As ATPases, they can transport the cation from the cytosol into the xylem sap against its electrochemical gradient. MRP transporters (ABCC) were responsible for the influx of PC-Cd complexes into the vacuole (Brunetti et al., 2015). Overall, we identified the major transporters related to Cd uptake and accumulation in the root of Zhe-Maidong, providing the putative genes for genetic improvement under Cd stress.

## 5 Conclusions

In this study, we evaluated the regularities of Cd transformation and distribution and developed a schematic model of Cd accumulation in Zhe-Maidong. The EF value in the root was four times that of the leaves, which shows that the root has a high ability to absorb and accumulate Cd. The Cd^2+^ distribution revealed that the epidermal cells of roots not only have the potential to accumulate Cd^2+^ but also have an excellent feature for Cd^2+^ uptake. *ANN, ABCC2/4, HMA1-5*, and *CCX* gene expression were positively correlated with EF-root, EF-leaf, EF-total, Cd-leaf, Cd-root, and Cd-plant, indicating their role in Cd transport and accumulation under Cd-stress. However, PDR3 and CDT gene expression were drastically upregulated in response to Cd stress, showing that they play a crucial role in avoiding Cd stress and promoting Cd tolerance. Our study lays the groundwork for future research into discovering novel genes and improving plant tolerance to Cd stress through selective breeding.

## Data availability statement

The original contributions presented in the study are publicly available. The raw sequence data reported in this paper have been deposited in the Genome Sequence Archive in National Genomics Data Center (Nucleic Acids Res 2022), China National Center for Bioinformation / Beijing Institute of Genomics, Chinese Academy of Sciences (GSA: CRA008254) that are publicly accessible at https://ngdc.cncb.ac.cn/gsa.

## Author contributions

Conceived and designed the experiments: QZ. Performed the experiments: QZ, QL, and SL. Analyzed the data: QZ, RH, JL, YT, YL, ZY, and XY. Wrote the paper: QZ, YZ, and JH. All authors contributed to the article and approved the submitted version.
