# Supplementary material for: De Novo transcriptome combined with physiological analyses revealed key genes for cadmium accumulation in Zhe-Maidong (Ophiopogon japonicus)
Source: Front Plant Sci. 2022 Dec 12;13:1078330. doi: 10.3389/fpls.2022.1078330 (PMC9791205; doi:10.3389/fpls.2022.1078330)
Supplement: Supplementary file 1 [file DataSheet_1.docx]

Supplementary Material

# Supplementary Figures and Tables

## Supplementary Figures

**Supplemental Fig. S1** **The correlation coefficient of the DEGs expression pattern obtained by RNA-seq and qRT-qPCR.**

**A.** Relative expression levels of seven DEGs under Cd stress as determined by qRT-PCR. **B.** Comparison between the log2 of gene expression ratios obtained from RNA-seq data and qRT-PCR. The qPCR log2 value of the expression (y-axis) was plotted against the value from the RNA-seq (x-axis). R^2^ represented the correlation coefficient between qRT-PCR and RNA-seq.

## Supplementary Tables

| Gene Name | ID | Forward Primer | Reverse Primer |
| --- | --- | --- | --- |
| GLR | Cluster-21637.128376 | ACGTGTGGAGGCGTTCCTGT | TGGAGGCTTGTGACGCGGTG |
| ANN | Cluster-21637.109116 | GTCACACGCTGCATCGCTTA | TGATCCAAGGGGATGCTGTT |
| PDR1 | Cluster-21637.9017 | GCTGCATCTTCCTCTGCTTTG | ATGGAGTTGAAGGTGGTCTCG |
| PDR2 | Cluster-21637.122135 | ATGAGCTCGCGAAGAAAGGCAT | TCGAATCAGTCTCCCCAGCAGCA |
| PDR3 | Cluster-21637.97527 | GGCCGCAAATGCAAAGAAAATGGGT | TGCCAAAGAAAGGGGCCGGA |
| CDT1 | Cluster-21637.48481 | GGCGAAAGAGAAGCCGGCCA | ACCGAGTAGGCGATCCGCGA |
| CDT2 | Cluster-21637.20982 | CGCCCATTCATCCCCTCCGC | CGAGGGTTGAGGGCTTCGGC |
| CAL1 | Cluster-21637.125183 | CAGTCCCCAGCTGGACCGTT | TCCAGAGGTGGCAGACATGGCA |
| CAL2 | Cluster-21637.16823 | AGGCCCGGGCACACTAGGAG | TCCCATCCCCTGCTGCTGCT |
| ABCC1 | Cluster-21637.124355 | TGATCCACCGCCTCCGTTGC | AAGGGAGCAGGCCCGGTGAA |
| ABCC2 | Cluster-21637.19887 | TGCCTAGCAGTCCACGTTGCAG | TCACTGCTATATACGCGCCCCCA |
| ABCC3 | Cluster-21637.124084 | TGCGAGAGGATGCGGTTGGC | TCCAATATGGGCAACAAAAGCCAAGA |
| ABCC4 | Cluster-21637.129096 | GCTGTGGAGTGCTGCGAGGG | GCCCGAACCCAAGAGCGACC |
| HMA1 | Cluster-21637.127989 | GGGGACGCTGATCCTGTAGA | TGAGCACATTGGAGGCAAGT |
| HMA2 | Cluster-21637.30363 | CCACATGGACTGCGATGGAT | GGGATATGGCCAGAACTCGG |
| HMA3 | Cluster-21637.18751 | CGTATTGTGGCCGTATCCGT | ACTCATCGTACCCGTGCTTC |
| HMA4 | Cluster-21637.115919 | GTCGAGCTCAAGGTGCGAT | CTATTTCAACGCAGGTGACTCC |
| HMA5 | Cluster-21637.19366 | TCAAGCTCCTCAAGATCCAGAC | TGCCAGAGACTGTCACCTTC |
| HMA6 | Cluster-21637.86821 | CAAGGGTGGCGAGAAGGGCG | CTGCGGCGGATGCACCATCT |
| HMA7 | Cluster-21637.58148 | AGAAGAAGACGGGCCGAAAG | CACTTTCAGCACCGCAACAA |
| ccx | Cluster-21637.16985 | CGTGCTGGGGGAGAATGGCG | GATGTCGTGCGACCCCGTCC |
| tubulin | Cluster-21637.82800 | CATCGATCACACCGGCAAGT | TCCAGATCTGACGGAGTCCA |

**Supplemental Table S1. Primers used in this study.**

**Supplemental Table S2. Clean reads of 9 samples.**

| Sample | Raw_reads | Raw_bases | Clean_reads | Clean_bases | Error_rate | Q20 | Q30 | Gc_pct |
| --- | --- | --- | --- | --- | --- | --- | --- | --- |
| Cd_0_1 | 23279350 | 7 | 22235218 | 6.7 | 0.03 | 97.37 | 92.93 | 48.51 |
| Cd_0_2 | 23848129 | 7.2 | 22918794 | 6.9 | 0.03 | 97.42 | 93.11 | 48.46 |
| Cd_0_3 | 24466902 | 7.3 | 23527884 | 7.1 | 0.03 | 97.59 | 93.5 | 48.47 |
| Cd_M_1 | 24610813 | 7.4 | 23364871 | 7 | 0.03 | 97.14 | 92.56 | 49.67 |
| Cd_M_2 | 24533000 | 7.4 | 23395722 | 7 | 0.03 | 97.17 | 92.77 | 49.33 |
| Cd_M_3 | 22847931 | 6.9 | 21864199 | 6.6 | 0.03 | 97.38 | 93.13 | 49.12 |
| Cd_H_1 | 23362920 | 7 | 22282920 | 6.7 | 0.03 | 97.43 | 93.24 | 49.35 |
| Cd_H_2 | 22944451 | 6.9 | 21784548 | 6.5 | 0.03 | 97.42 | 93.24 | 48.99 |
| Cd_H_3 | 22663878 | 6.8 | 21516690 | 6.5 | 0.03 | 97.5 | 93.42 | 48.72 |
